# Pain Symptomatology and Management in Pediatric Ehlers–Danlos Syndrome: A Review

**DOI:** 10.3390/children7090146

**Published:** 2020-09-21

**Authors:** Estée C. H. Feldman, Daniel P. Hivick, P. Maxwell Slepian, Susan T. Tran, Pradeep Chopra, Rachel Neff Greenley

**Affiliations:** 1 Department of Psychology, Rosalind Franklin University of Medicine and Science, North Chicago, IL 60064, USA; rachel.greenley@rosalindfranklin.edu; 2 Chicago Medical School, Rosalind Franklin University of Medicine and Science, North Chicago, IL 60064, USA; daniel.hivick@my.rfums.org; 3 Toronto General Hospital, University Health Network, Toronto, ON M5G 2C4, Canada; maxwell.slepian@uhn.ca; 4 Department of Psychology, DePaul University, Chicago, IL 60614, USA; susan.tran@depaul.edu; 5 Alpert Medical School, Brown University, Providence, RI 02903, USA; painri@yahoo.com

**Keywords:** Ehlers–Danlos syndrome, chronic pain, management of pain, musculoskeletal pain, quality of life, joint hypermobility syndrome, adolescent, emerging adulthood

## Abstract

Ehlers–Danlos syndromes (EDS) are a group of connective tissue disorders that manifest with hyperextensibility of joints and skin, and general tissue fragility. While not a major criterion for clinical diagnosis, pain is a frequently endorsed symptom across subtypes of EDS. As such, the present review aims to summarize research to date on pain characteristics and management, and the relationship between such pain symptomatology and quality of life in pediatric EDS. Characteristics of pain, including theorized etiology, relative intensity and extent of pain are described, as well as descriptions of frequently endorsed pain sites (musculoskeletal, and non-musculoskeletal). Interventions related to the management of musculoskeletal (e.g., pharmaceutical intervention, physical therapy) and non-musculoskeletal pain (e.g., pharmaceutical and psychological interventions) are discussed, highlighting the need for additional research related to pediatric pain management in the context of hypermobility syndromes. In addition, the relationship between pain in pediatric EDS and quality of life is described. Finally, limitations of literature to date are described and recommendations for future lines of research are outlined.

## 1. Introduction

The Ehlers–Danlos syndromes (EDS) encompass a heterogeneous group of incurable connective tissue disorders that are clinically characterized by skin hyperextensibility, joint hypermobility, subluxation and dislocation, and general tissue and vasculature fragility [1,2]. According to the most recent classification system, described by Malfait and colleagues, 13 subtypes of EDS are presently described. The hypermobility type (hEDS), followed by the classical type of EDS present most commonly, together accounting for over 90% of cases [3]. Presently, gene mutations and related genetic testing have been identified and utilized for diagnosis of the classical, arthrochalasia, kyphoscoliosis and vascular forms of EDS [4]. However, no genetic testing is available for hEDS, and the mutations and mode of genetic transmission remain poorly understood. Some theorize a sex-linked inheritance pattern, given that there are more female patients are diagnosed with hEDS than males, especially within hEDS [5,6]; however, underdiagnosis of male patients may also account for this imbalance. Given the lack of genetic testing, diagnosis of hEDS is based on clinical examination (e.g., classification delineated by Malfait and colleagues [7]) and family history [2]. With respect to the three most common forms of EDS (hypermobility, classical, vascular) focused on in the present review, all three present with proposed autosomal dominant inheritance, and share several diagnostic features. However, while major diagnostic criteria for vEDS are marked by severe cardiovascular and valvular consequences (e.g., arterial rupture at young age, uterine rupture during pregnancy, spontaneous colon perforation), cEDS is distinguished by skin hyperextensibility, scarring and generalized joint hypermobility, and hEDS is marked by generalized joint hypermobility, positive family history of hEDS, and the exclusion of diagnostic criteria associated with other forms of EDS [7].

Despite the heterogeneity of subtypes and associated symptoms, pain symptomatology is commonly endorsed across all subtypes of EDS [7]. There are numerous causes of pain in EDS, including hypermobility of joints, increased frequency of subluxation and dislocations of major and minor joints, injury to soft tissue and muscle pain, and prior surgical intervention [8]. Often, pain in EDS becomes chronic [9,10], leading to increased functional disability [3] and poorer psychological health [11] in adult patients [12]. 

However, while the onset of symptoms of EDS occurs as early as the first year of life [13], individuals with EDS often do not receive a diagnosis until adolescence or emerging adulthood [14] with delays of several years between symptom onset and intervention [15], making the study of symptomatology in pediatric populations challenging. As such, little is known about how the aforementioned pain symptomatology and diminished quality of life described in adults may generalize to pediatric and emerging adult populations. Yet, research that describes pain symptomatology and related interventions in this demographic group is crucial, given that findings from other pediatric populations highlight how the experience of chronic pain early in life is associated with poorer quality of life and continued chronic pain in adulthood [16]. Therefore, the present review aims to describe pain characteristics in pediatric and young adult populations with various forms of EDS, describing known interventions to manage such pain as well as the relationship between pain symptomatology and quality of life in the context of pediatric EDS. 

Notably, given the relative lack of research in samples with pediatric EDS specifically, studies commonly include pediatric joint hypermobility syndrome (JHS) patients, as several experts consider JHS and hEDS to be clinically equivalent [17,18,19]. Like those with hEDS, individuals with JHS are diagnosed based on hypermobility criteria (e.g., Malfait classification) and on the presence of arthralgia (though, a “minor” rather than a “major” diagnostic criterion for hEDS) [19,20]. As such, the present review reports on pain characteristics and treatments, and quality of life as it pertains to pain in the context of pediatric JHS as well as EDS.

## 2. Pain Characteristics

### 2.1. Etiology of Pain 

Despite being a widely reported symptom in EDS and JHS, with these pediatric populations reporting significantly more intense pain than the general population [21], the etiology of pain in hypermobile individuals (i.e., those with JHS and EDS) remains poorly understood [22].

One theory posits that repetitive soft tissue microtrauma is the major mechanism by which joint pain develops, especially in the lower body [23]. This theory proposes that the hyperextension of weight-bearing joints coupled with the lack of proprioceptive acuity and reduced muscle strength and endurance by which to control movement noted in hypermobile individuals [24] leads to microtrauma and abnormal loading on joints, resulting in pain [25]. Notably, while microtrauma is present in individuals with and without EDS, as a result of the balance between tissue damage and repair, in individuals with EDS, the tissue is damaged more quickly than it can be repaired. This imbalance results in more extensive damage with slower repair in patients with EDS than is seen in the general population, as exemplified by findings from several case studies [26,27]. Similarly, the aforementioned lack of proprioceptive acuity fosters the development of microtrauma by failing to protect from hyperextension. Furthermore, both microtrauma and macro-trauma (e.g., ligament injuries, subluxations, dislocations) in turn diminish proprioceptive abilities, increasing the likelihood of further injury and pain [28]. 

Other theorists propose that the generalized joint instability characteristic of EDS fosters the development of microtraumas in joints which cause adaptation of movement patterns, in turn leading to overload in other areas of the musculoskeletal system [29] regardless of whether or not they are weight bearing. However, neither of the previously mentioned theories readily account for pain in non-musculoskeletal systems, and no empirical support for either has been tenable. 

While most suggest that the etiology of musculoskeletal pain in EDS is rooted in the hyperextensibility characteristic of the syndrome [23,24,29] others propose that the chronic widespread pain, beyond the musculoskeletal system, endorsed in this population is attributable to generalized hyperalgesia, increased sensitivity to pain [30]. An examination of pain pressure thresholds (PPTs) at symptomatic and asymptomatic sites (as indicated by responses on a Margolis Pain Diagram) between adult women with hEDS and age and gender-matched healthy controls suggested that, compared to controls, those with hEDS indicated significantly lower PPTs at both painful and pain-free body locations [30]. The authors conclude that said findings provide evidence for secondary hyperalgesia among those with hEDS, suggesting the presence of a highly sensitized central nervous system as a central mechanism underlying chronic pain in this population [30]. However, given their lack of consideration of and controlling for covariates (e.g., prior injury, frequency of dislocations and subluxations, and hyperextensibility of joints in question), as well as their sex-restricted (i.e., female only), hEDS specific, and relatively small (N = 23) sample size, such conclusions presently lack adequate support. Therefore, a study that considers such covariates in a more diverse population of patients with EDS, ideally with pediatric patients, is needed prior to reaching conclusions about the role of hyperalgesia as the mechanism underlying chronic pain in pediatric EDS. 

**Figure 1:**
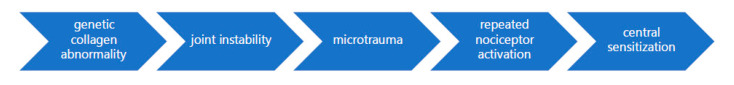
Integration of theories of etiology of pain in Ehlers–Danlos syndromes (EDS) described by Gedalia and Brewer (1993) [23], Ferrell and colleagues (2004) [29], and Rombaut and colleagues (2015) [30].

Therefore, presently, no one theorem of the etiology of EDS can account for the multiple forms of pain frequently endorsed by this population. However, the etiology of pain described by the aforementioned theories are not mutually exclusive. For example, if central sensitization is indeed present, it may be attributable to the marked history of injury and microtrauma, as repeated nociceptor activation is a prerequisite for central sensitization [31,32]. As such, given that said theories are likely to be interdependent, a cohesive description of the etiology of pain in EDS is described in Figure 1. 

### 2.2. Pain Intensity and Extent 

As previously mentioned, pediatric EDS and JHS populations report greater pain intensity than healthy age-matched controls [21] as well as numerous locations of pain [30,33]. Recently, a study of youth (aged 8–17 years old) with hEDS reported on the intensity and extent of pain [33]. On a valid and reliable assessment of children’s pain intensity [34], with an 11 point numeric rating scale, ranging from “no pain” (0) to “worst pain I can imagine” (10), respondents indicated an average pain intensity of 5.53 (SD = 2.6), indicating on average moderate pain. Similarly, on the Widespread Pain Index (WPI) a 19-item inventory indicating the occurrence of pain in 19 defined body locations, youth indicated pain in an average of 7.06 locations (SD = 5.07) [33]. The widespread nature of this pain is notable, as a WPI score greater than 7 (coupled with a Symptom Severity Score greater than 5, also endorsed by the aforementioned sample) is considered clinically indicative of a diagnosis of fibromyalgia [35]. Such findings highlight how extensive and intrusive pain symptomatology in pediatric EDS can be, essentially replicating the painful features of other chronic pain syndromes (e.g., fibromyalgia). 

### 2.3. Sites of Pain: Musculoskeletal

Musculoskeletal pain sites in JHS and EDS have been well reported on, with several studies describing the joints reported as most impacted by pain in pediatric populations. In a recent study of children and adolescents with hEDS [33], the most commonly reported pain locations were the lower back, neck and shoulders, with over half the sample reporting pain in each of the aforementioned sites. Similarly, findings from a retrospective medical record review of youth (ages 6 through 19) with a variety of types of EDS (55.6% hEDS) indicated that the most common sites of presenting pain were the knee (43.4%), back (32.2%) and shoulder (31.2%) [36]. Similarly, children with previously diagnosed JHS or hEDS, indicated, through marking on a body drawing where they experienced pain, the greatest pain in their feet and knees, followed by their shoulders and wrists [21]. Notably, children without any hypermobility syndrome in the control group did not report pain in any bodily site more than another, only reporting muscle aches or pain secondary to minor accidents [21]. Among samples of children with a diagnosis of JHS, such widespread pain is similarly endorsed. Eighty-eight percent of sampled children with JHS endorsed pain, specifying pain in their feet (64%), knees (61%), back (44%), hips (38%), and neck (32%) [37]. Widespread musculoskeletal pain is noted in several subtypes of EDS. For example, longstanding back and neck pain are both reported in a case study of a 15-year-old with classical EDS [38]. While present in other forms of EDS, the study of musculoskeletal pain has been heavily focused on those with hEDS and JHS, likely due to the relative ease of forming a substantial size necessary for analysis, and potentially reflecting the lower incidence of painful symptomatology in other subtypes. As such, future research examining the occurrence of musculoskeletal pain extending beyond case studies in other forms of pediatric EDS is required. 

While the aforementioned studies highlight pain in major, often weight-bearing, joints consistent with theories of previously mentioned theories of musculoskeletal pain etiology [25], pain in smaller joints is also noted in pediatric EDS populations. For example, in a case study of two male siblings with hEDS, joint pain was endorsed in the fingers, toes and wrists, as well as in larger, joints such as ankles, knees, elbows and shoulders [39]. Similarly, two other case studies of children with hEDS also indicated pain in the fingers, specifically due to painful subluxation of the carpometacarpal joint of the thumb [40]. Such pain in smaller joints has also been reported among older adolescents with EDS, wherein a case study of an 18-year-old woman with hEDS indicated substantial finger pain, specifically of the thumb due to frequent dislocations [41]. Pain in smaller joints is also reported in children with JHS, with 32% indicating wrist pain [37]. While limited in scope (i.e., specific to those with hEDS), such case studies suggest that pain in smaller joints may be attributable to more frequent subluxations, secondary to greater wakening of ligamentous constraints in such joints [40]. The authors refer you to Table 1 for a summary of musculoskeletal pain sites described in pediatric hypermobility syndromes. 

### 2.4. Sites of Pain: Non-Musculoskeletal 

While musculoskeletal pain is often the form of pain referenced in discussions of and research dedicated to EDS, in pediatric populations with JHS and EDS, headaches, abdominal pain, dysmenorrhea, and rare, painful podiatric pathology (e.g., painful piezogenic pedal papules) are also documented.

Headaches present with relative frequency among hypermobile pediatric patients, with 40% of children with JHS presenting with headaches [33,37]. Additionally, headaches are described in several case studies of children with classical EDS. In a case study of a 15-year-old female patient with classical EDS, a 10-year history of intractable, migraine headaches were endorsed [38]. She described tri-weekly migraine headaches, accompanied by photophobia, phonophobia, nausea and vomiting, as well as tension-type headaches. Further, in pediatric classical type EDS populations, headaches have also been reported in the context of subependymal periventricular heterotopias. Subependymal periventricular heterotopias describe a developmental cortical malformation marked by masses of neurons that fail to properly migrate during central nervous system development and instead form nodules of grey matter that inappropriately line the lateral ventricles, deep to the ependymal cell layer. This malformation can present in a variety of ways, from clinical silence to recurrent partial or complex–partial seizures and even intellectual disability [47]. Despite the rare relation to headaches and migrainous pathology, three case studies of adolescent females with classical type EDS and subependymal periventricular heterotopias, all endorsed related headaches [42]. While literature remains relatively sparse in pediatric populations, amongst adults, approximately half of patients with EDS report headache, of poorly understood pathophysiology [9]. Such studies indicate that among adults with EDS, frequently endorsed forms of headaches include migraines with and without aura, tension-type, combination of migraine and tension-type [48], and cerebrospinal fluid leak headaches [49]. In fact, in adults with EDS, such leakage and associated headaches often present as initial symptoms of underlying connective tissue pathology [49]. However, in pediatric populations, most examinations of headaches and associated pathology are restricted to the classical form of EDS and occur in the context of case studies, or are described solely in the context of JHS. Further investigation is warranted to determine the frequency and nature of such pathology in populations with other forms of EDS. 

Similarly, additional research is needed to address the occurrence and nature of abdominal pain in such pediatric populations. While the mechanism underlying gastrointestinal involvement and related pain in EDS remains poorly understood [50], in the few studies which have investigated gastrointestinal complaints, abdominal pain presents as a source of notable discomfort among children with EDS and JHS. Among children with JHS, 35% indicate abdominal pain as a source of significant pain [37]. Similarly, a more recent study indicates that 54% of children and adolescents with JHS and hEDS endorsed gastrointestinal complaints, including abdominal pain [17]. Notably, prior research indicates significant comorbidity between hypermobility syndromes and pain-related functional gastrointestinal disease (FGID). A study of children and adolescents with various pain-related FGID such as irritable bowel syndrome (IBS), functional dyspepsia, functional abdominal pain and abdominal migraine, indicated that over half of subjects also met full diagnostic criteria for JHS, a significantly greater percentage than is present in the general population [51]. Despite such findings, many note the relative dearth of literature related to abdominal pain and hypermobility syndromes in pediatric populations, despite significant evidence in adult populations suggesting this line of inquiry necessitates study. As early as 1969, “recurrent, vague, ill-defined”, “severe” and “excruciating” abdominal pain [52] has been reported in adults with various forms of EDS. More recently, among adults with hEDS, painful gastrointestinal complaints including abdominal pain, constipation, nausea and diarrhea [53] were commonly endorsed and believed to be attributable to dysautonomia often endorsed by this population [53,54]. Similarly, in another sample of 21 adult hEDS patients, 62% indicated recurrent abdominal pain, 67% endorsed dyspepsia (painful indigestion) and 57% reported gastroesophageal reflux [55], suggesting that while not a diagnostic criterion, abdominal pain is a common complaint in the context of hEDS. While the aforementioned research reports on studies of hEDS participants, abdominal pain remains the most common GI symptom when comparing EDS types to one another. Abdominal pain was endorsed by 51.2% of adults with classical EDS, 56.1% of those with hEDS, 81.5% of those with vascular type EDS, and 43.2% of those with other forms of EDS [56], indicating that regardless of the type of EDS, abdominal pain is likely to present as a painful symptom necessitating management. Given the frequency of the endorsement of abdominal pain in adults with EDS generally, with upwards of half of all adults with hEDS meeting Rome IV criteria for the diagnosis of FGID [57], coupled with the comorbidity of hypermobility syndromes and FGID noted in pediatric samples, further examination of such symptomatology in pediatric EDS populations is warranted. The lack of evidence examining the frequency and nature of abdominal pain complaints in pediatric EDS samples is a considerable shortcoming of research to date, given that crucial, life-threatening differential diagnoses to consider with abdominal pain presentation include aneurysm, rupture, dissection, varicosities and arteriovenous fistula formation [58]. 

For females with EDS, pain has also been described in the context of the menstrual cycle. In a retrospective chart review of 156 adolescent females with both vascular type and hEDS, over half reported dysmenorrhea (i.e., painful menses), a considerable decrease compared to rates of dysmenorrhea in adult females with EDS (72–93% [43,44]). While rates of dysmenorrhea in youth with EDS are comparable to rates of moderate dysmenorrhea described by healthy adolescents, (43–93% [44]), other research indicates that between 5 to 23% of healthy adolescent females report severe dysmenorrhea [59]. Therefore, future research within pediatric EDS which considers the frequency with which more intrusive, severe dysmenorrhea is endorsed would be valuable. Furthermore, given that pain presents as a primary gynecological complaint (e.g., vulvodynia, vestibulodynia, dyspareunia, and generalized pelvic pain [60]) in adult females with EDS, the greater study of such pain, beyond dysmenorrhea, is warranted to facilitate earlier and more effective intervention. 

While in adults with EDS, painful piezogenic pedal papules are cited as a cutaneous manifestation of EDS, seen in over 1/3 of patients [61] such symptomatology is less frequently described in the pediatric population [45]. Piezogenic pedal papules are herniations of subcutaneous fat through the dermis of the foot which appear and cause notable discomfort when weight-bearing [62] and are believed to be due to structural defects of the connective tissue seen in those with EDS [45]. First described in a pediatric case study in 1985, a 5-year-old female child with EDS endorsed substantial pain subsequent to such herniations on the medial and lateral aspects of her feet upon standing [45]. Similarly, a more recent case study of a 2-year-old female child (as well as her father) with classical EDS [46] similarly indicates the appearance of these painful papules upon walking. Notably, as such podiatric pathology is uncommon in toddlers in the general population, the presence of such papules may serve as a diagnostic “red flag” for EDS in this age group [46].

As evidenced by the relative dearth of literature addressing non-musculoskeletal pain complaints in the pediatric EDS population, additional research is needed to determine whether the painful symptomatology reported in such systems in adults EDS populations is similarly endorsed in a younger demographic group and whether such symptoms are as prevalent as the well-reported musculoskeletal pain. For example, among adult populations with EDS, other comorbid conditions have been documented to modulate pain and the pain response. Such conditions include Chiari malformations, craniocervical instability, tethered cord syndrome [63], dysautonomia [2], postural orthostatic tachycardia syndrome (POTS) and mast cell activation syndrome [64]. Given that the aforementioned conditions have been associated with a high incidence of co-morbidity in children with EDS [65] further research which considers the effects of such comorbidities on pain is warranted. The authors refer you to Table 1 for a summary of non-musculoskeletal pain sites described in pediatric hypermobility syndromes. 

## 3. Pain Management Interventions

### 3.1. Interventions for Musculoskeletal Pain

The aforementioned lack of understanding as to the etiology of musculoskeletal pain in EDS and JHS makes the linkage of intervention with theory challenging [14]. As such, while current expert opinion is that physical therapy should be the first line of treatment for musculoskeletal pain of suspected hypermobile etiology [66], musculoskeletal pain management recommendations and the consistency with which they are implemented in pediatric populations vary. For example, while in a sample of children and adolescents with EDS (predominately hEDS, 56% [36]) the majority (88.1%) were prescribed physical therapy to manage their musculoskeletal pain, a considerable portion was prescribed rest (40.5%), orthotics (35.6%), medication (32.2%) and even surgery (28.8%). Similarly, in a study of children with JHS [37] participants endorsed having utilized pharmaceutical pain relief (85%), podiatry (42%), insoles (35%), exercises (33%), stretching (12%), occupational therapy (13%), weights (6%), and hydrotherapy (4%), indicating that despite expert recommendations, physical therapy remained underutilized. 

However, despite being the recommended first line of treatment for musculoskeletal pain in the context of hypermobility in pediatric EDS and JHS, outcomes of specialized physical therapy interventions have been mixed. In the first randomized control trial (RCT) of physical therapy for the treatment of hypermobility-related pain, children and adolescents with JHS were either assigned to a targeted or general physical therapy program for 6 weeks with the goal of reducing their pain, increasing their functional status and improving their fitness [67]. The targeted exercise program focused on controlling their neutral joint position (e.g., avoiding hyperextension of the knee when standing), retraining dynamic control (e.g., hip flexion while maintaining a neutral spine), developing motion control (e.g., working concentrically on standing up and eccentrically on sitting down) and specific tissue lengthening (e.g., hamstring stretches), while the general program focused on more global exercises designed to increase strength (e.g., shuttle runs, step-ups). While both groups showed improvements in pain as a function of the intervention, no between-group differences were detected, suggesting that physical therapy designed for hypermobility is no more effective than general intervention. Since the initial RCT, other physical therapy-based interventions have been designed to evaluate the efficacy of physical therapy programs created for hypermobility and yielded similar results. A prospective, parallel-group RCT of an 8-week exercise program for children (age 7–16) with JHS and related knee pain similarly studied the efficacy of a program focused on improving strength and control around the knee joint to reduce knee pain through exercises in the hypermobile range versus in neutral knee extension [25]. However, as in the original RCT [67], while both groups presented with diminished maximal knee pain as a function of the intervention, no differences were detected between the general and specialized physical therapy groups. Therefore, while physical therapy interventions designed specifically for the hypermobile range appear to be no more effective than general physical therapy, given that general interventions remain successful in reducing musculoskeletal pain in pediatric hypermobility syndromes and carry few risks, they remain the optimal starting point of intervention for pediatric populations endorsing pain in the context of hypermobility. 

As previously indicated, in addition to, or at times in place of, physical therapy, a substantial percentage (32.2–85% [36,37]) of pediatric EDS and JHS patients have been prescribed pharmaceutical interventions to manage musculoskeletal pain. Case studies to date indicate that oral tramadol, an opioid analgesic, is effective in reducing such pain in pediatric hEDS patients. In fact, case studies offer preliminary evidence for the efficacy of tramadol for the management of musculoskeletal pain even for very young patients (e.g., as young as 3.5 years old) with hEDS [39]. Such promising findings are consistent with findings of efficacy for treatment of chronic pain in pediatric populations more generally, which indicate that the use of 50mg tramadol administered orally every 4–6 h for 7–30 days has been well-tolerated in children (ages 7–16) with a variety of painful conditions, and deemed successful in reducing pain by 69% of parents [68]. However, as no RCT has been conducted on the efficacy of tramadol in the context of pediatric chronic pain generally, or JHS and EDS more specifically, and that it is not FDA-approved for use in pediatric populations [69], conclusions regarding the efficacy and long-term safety of such intervention are not presently tenable. Given that, in adults with EDS, risks of tramadol include worsening common gastrointestinal complaints such as nausea and constipation, as well as aggravating symptoms of the frequently co-occurring mast cell activation syndrome (MCAS) [8], consideration of their safety for use in the pediatric population is warranted.

While relative success has been described in both pharmaceutical intervention (e.g., tramadol) and physical therapy, other attempted interventions to manage musculoskeletal pain have been less successful. In a study of four 9th grade students with JHS who presented with handwriting difficulty secondary to wrist pain, the use of a wrist brace was implemented [70]. However, there was not a significant decrease in pain with the use of the brace; in fact, 75% of students reported significant pain from the brace itself, only to have that pain relieved upon withdrawal of the splint. 

As evidenced by the variety of interventions described above, recommendations for the management of musculoskeletal pain in the context of pediatric hypermobility syndromes are variable, likely due to the poorly understood etiology of such pain, as well as the sparsity of RCTs which assess the efficacy and safety of each of the aforementioned interventions. In order to provide more evidence-based, systematic recommendations for the management of musculoskeletal pain in this population, a greater understanding of the etiology of such pain must be developed and RCTs examining the efficacy of more carefully designed physical therapy and pharmaceutical interventions must be conducted. 

### 3.2. Interventions for Non-Musculoskeletal Pain

As the majority of research which describes pain in pediatric JHS and EDS has focused on the chief pain complaint of musculoskeletal pain, so too has research related to the management of said pain. As such, recommendations specifically for pediatric patients suffering from headaches, dysmenorrhea, or painful piezogenic pedal papules are limited. However, recommendations for the occurrence of such symptoms in an adult cohort of patients with EDS, and age-matched healthy peers, have often been noted, and may be appropriately tailored for this particular population. 

Regarding headaches, a case study has reported on the treatment of migraine and tension-type headaches in the context of pediatric EDS. In the case of the 15-year-old female with classical type EDS with the decade long history of headaches, following MRI with non-significant findings, a trial of magnesium was implemented, though the outcomes were not reported [38]. Such a recommendation avoids the use of non-steroidal anti-inflammatory drugs (NSAIDs), which, in adults with EDS yields adverse gastrointestinal, renal and hematologic side effects [9], and may exacerbate the symptoms of co-morbid MCAS [71]. However, as literature in pediatric EDS is limited, it should be noted that in the adult population, headaches are treated based on suspected etiology of pain. For example, headaches resulting from neck pain secondary to loose ligaments in the cranio-cervical junction and cervical spine [72] might be treated with physical therapy or exercise interventions to indirectly reduce head pain. However, migraine headaches and tension-type headaches might be treated pharmaceutically (e.g., acetaminophen to avoid the hematologic consequences associated with NSAIDs [8]). Therefore, to better guide the treatment of headaches in pediatric EDS, a greater study of the causes of such pain must first be conducted in order to guide recommendations for treatment. 

As with the treatment of headaches, because abdominal pain itself has received inadequate attention in pediatric EDS samples, recommendations related to the management of abdominal pain in this demographic group remain similarly limited. Despite abdominal pain being well reported in adults with EDS, no research which identifies interventions to alleviate such uncomfortable symptoms outside a concurrent diagnosis of FGID could be identified, likely due to the unclear etiology of such pain in the context of EDS. However, given that the frequency with which adults with EDS are diagnosed with FGID, treatment related to such diseases may be appropriate to reduce pain in the context of FGID comorbid with EDS in pediatric samples. To date, various psychological interventions, such as cognitive-behavioral therapy (CBT) and hypnosis [73] have successfully produced significant reductions in painful FGID symptoms among pediatric populations. Pharmaceutically, the use of peppermint oil and cyproheptadine, a first-generation antihistamine, was found to reduce the frequency and intensity of pain in children and adolescents with abdominal pain-related FGID [74]. While such findings are promising for pediatric EDS samples with comorbid FGID, greater investigation and intervention development are needed to adequately address the symptoms of pediatric EDS patients who experience abdominal pain without reaching the diagnostic threshold for the diagnosis of another condition, such as an FGID. Additionally, for those who experience abdominal pain, especially that related to limited GI motility, the use of opioids to manage chronic musculoskeletal pain may be ill-advised. As the use of opioids further reduces the already compromised gastric motility [75] noted in patients with EDS, increasing associated pain [75], patients who experience concurrent abdominal and musculoskeletal pain may be advised to utilize another treatment modality to address their musculoskeletal pain, as outlined above. 

While recommendations related to gynecological pain in the context of pediatric EDS remain similarly limited, interventions are described for adults with EDS and adolescent females with dysmenorrhea in the general public. Given its time-limited nature, dysmenorrhea in pediatric EDS patients can be safely pharmaceutically treated with NSAIDS without overt risk of hematologic complications [8,43,76]. Additionally, prior research on adolescent females without EDS indicates that the use of an oral contraceptive (ethinyl estradiol and levonorgestrel) over the course of 3 months led to significant reductions in menstrual pain [59]. Similarly, in adult females with EDS, hormonal control intervention has been successful in reducing dysmenorrhea [43]. Given the success in adult populations with EDS, and adolescent females more generally, the use of hormonal intervention may be a promising point of intervention for the treatment of dysmenorrhea in adolescent females with EDS. 

Finally, while treatment of painful piezogenic pedal papules has not been described in pediatric EDS populations specifically, in adults with EDS, successful intervention has been described and may be adapted for younger populations. In an adult male with painful piezogenic pedal papules in hEDS, three injections of a solution of equal parts betamethasone and bupivacaine over the course of 3 months were curative and effects were sustained over a 5-year course [77]. In addition, a combination of a steroid and anesthetic injection has been described as another method of treatment in the management of painful piezogenic pedal papules [77]. More recently, painful piezogenic pedal papules have been effectively treated via single injection lipolysis using deoxycholic acid (DCA) [78]. While no case studies or efficacy trials have yet to be conducted in pediatric populations, likely due to the relative infrequency of such pathology in this demographic, such minimally invasive interventions are similarly likely to be effective in reducing pain due to piezogenic pedal papules in pediatric EDS populations. 

## 4. Pain and Quality of Life in Pediatric EDS 

Decreased quality of life, as measured by psychosocial, emotional, physical and school functioning has been relatively well documented in those with EDS, with diminished functioning noted in adult [79,80,81] and pediatric [82,83] populations alike. While many propose that this diminished quality of life is explained by the increased frequency and intensity of pain experienced by the population as compared to the general public [84], few lines of inquiry have directly assessed this theory. However, the little research which has examined this relationship provides evidence in support of the proposal. The authors refer you to Table 2 for a brief summary of the quality of life correlation of pain in hypermobility syndromes, which are expanded upon in more detail presently. With respect to emotional functioning, among children and adolescents with EDS, greater intensity of pain, as well as a greater number of pain sites was associated with greater anxiety and depression [33]. Similarly, among children with JHS for whom pain was a significant symptom (88%), only 35% indicated being able to attend school full time, suggesting that the ability to attend school is further diminished among children with EDS who endorse pain [37]. 

Similarly, deficits in physical functioning have been noted in pediatric JHS and EDS populations who endorse pain. Both the number of pain sites and pain intensity were positively associated with greater functional disability in children and adolescents with hEDS, and only 6% of children with pain symptomatology in the context of JHS reported completing activities in their school gym classes [37]. Further, children with JHS present with lower maximal exercise capacity, compared to age- and gender-matched control subjects, as measured by diminished peak maximal oxygen consumption [85]. Researchers propose that the relatively poorer aerobic fitness noted in children with JHS is due to their experience of musculoskeletal pain, which leads to inactivity and subsequent deconditioning [85]. The relationship between pain and physical functioning is further complicated by reports of pain exacerbation through activity in youth with JHS [15]. Notably, 81% of children and adolescents with JHS indicate that their pain is exacerbated by exercise, with 65% indicating pain immediately following activity, 59% reporting pain later in the evening and 50% indicating pain the following day [15]. Given the increase in pain following exercise, and that exercise (i.e., physical therapy) is the recommended modality to manage musculoskeletal pain in the context of hypermobility syndromes, providers should consider this when making recommendations to patients. Providers may ask about the prior exercise experiences of pediatric JHS and EDS patients and adjust their explanation of why physical therapy is recommended accordingly. Providers may need to explain that exercises can be adapted to manage pain and prevent injury to reduce the perception of exercise as potentially frightening or inaccessible. Moreover, such conversations would be crucial to addressing kinesiophobia (fear of pain, movement or re-injury) which may develop as a function of the chronic pain experienced by populations with EDS [82]. 

Although some research to date, as above described, has delineated a relationship between pain and metrics of quality of life in pediatric JHS and EDS, a greater examination of as-of-yet under-examined aspects (e.g., social functioning) is warranted. This line of inquiry is especially valuable, in light of recent findings which suggest that among the predictors of quality of life in pediatric EDS, greater pain, along with greater fatigue, presents as the strongest predictor of diminished quality of life [83]. Through horseshoe and elastic net regressions, when selecting from various demographic and disease variables, pain and fatigue emerged as the strongest predictors of every measure of quality of life (physical, emotional, social, school, and psychosocial functioning) among adolescents with hEDS [83]. As such, a greater examination of how pain impacts quality of life in pediatric EDS is warranted. Future research may be advised to consider how pain-related variables, such as the number of pain sites, duration of pain, and even qualitative description of pain relate to self-reported quality of life. 

## 5. Limitations of Research and Recommended Future Directions

Research to date, however informative, must be interpreted in light of several shortcomings in sample construction. Classified as a rare condition, acquiring an adequately sized sample of participants (especially within a restricted age range) with EDS poses a challenge. As such, it is not uncommon for articles to either generalize their findings to all types of EDS based on a sample of hEDS (or even JHS) participants, or to include a sample of mixed types of EDS without considering or controlling for potential differences between types. However, given that certain symptoms are more common in certain types of EDS, findings in one type may not generalize to others, and examining the many types as one group may disguise unique variance in symptomatology between types. For example, individuals with hEDS indicate a greater prevalence of pain as compared to those with classical type EDS, while those with hEDS and classical type EDS indicate greater present pain than those with vascular type EDS [3]. As such, collapsing to create a single EDS group may conceal significant differences in pain symptomatology between types. Furthermore, given differences in symptoms endorsed between groups, the nature of pain itself may differ, with patients with some forms of EDS endorsing more chronic pain, and others experiencing more severe, acute pain. While the present review is limited in its focus on chronic pain in this population, as findings to date indicate the differential experience of pain (i.e., acute versus chronic) as a function of the EDS subtype, further study is encouraged. For example, those with vascular type EDS are at greater risk of aortic disease as compared to those with classical type and hEDS [85,86]. As aortic pathology involves compromised blood flow to internal organs and extremities, resulting in acute pain, those with vascular type EDS may not experience chronic pain with as much intensity, but may have greater instances of severe, unpredictable, acute pain, which may be life-threatening. Given such potential differences, qualitative research that captures underlying differences in the nature of pain in different forms of EDS would be valuable to help educate treatment plans as well as determine the extent to which generalizability between EDS types is appropriate. 

The present findings are further limited in their generalizability given shortcomings with respect to demographic diversity in sample construction. While hEDS is known to, for reasons poorly understood, disproportionately affect females [87,88], the predominance of female studies of hEDS limit generalizability to males with hEDS, and others with EDS more generally. Studies describing pediatric EDS populations presently summarized have samples ranging from 71% female [33] to 100% female [89] leaving little room to adequately describe or account for pain symptomatology in men with various types of EDS. While findings related to females are informative, given known differences in both the experience and response to the management of pain between sexes rooted in biological (e.g., sex hormones influence on pain sensitivity) and psychosocial processes (e.g., pain coping, early life exposure to stress, stereotypical gender role ascription) alike [90] generalizations to the experience of males is ill-advised. Notably, however, samples composed of children and adolescents with JHS [15,37,86], or EDS and JHS [17,18,21] present a much more balanced sample with respect to sex, indicating ranges of female representation ranging from the minority (38% [84]) to the slight majority (66% [37]). If representative of true differences between the diagnostic groups, greater caution is recommended in further equating the clinical diagnoses of JHS and hEDS. 

In addition to the lack of representation and consideration of males with EDS, generalizations are ill-advised with respect to race and ethnicity, given the predominant focus on White, non-Latinx, children and adolescents with JHS and EDS. To date, the majority of studies indicate a White non-Latinx subject majority, ranging from 64% [33] to 93.6% [83] with most studies falling in the upper range. It should, however, be noted that such a lack of representation in research is not believed to be representative of the true rate of incidence of EDS in more racially diverse populations, but instead the pervasive racial bias in the assessment and treatment of pain [89] leading to underdiagnosis of EDS in said populations. Prior research finds that medical students and residents hold false beliefs about biological differences between Black and White people with respect to their perception of pain and need for treatment to manage said pain [91]. Such biases in turn lead to the underestimation of and undertreatment of pain amongst pediatric [92] and adult Black populations [93,94], as well as in Latinx individuals [95]. Pain dismissal of Black and Latinx children and adolescents may then lead to a lack of investigation of such symptoms, which in turn may preclude an appropriate diagnosis of EDS. Therefore, the biased nature of assessment of and response to the pain of Black and Latinx children and adolescents may lead to inappropriate underdiagnosis of EDS in these communities. Given such potential underdiagnoses, and society-wide differences in perception and treatment of pain in Black and Latinx pediatric populations, findings to date are unlikely to adequately represent the experience of said populations. As such, additional research examining pain and pain management in pediatric EDS in the context of racial minority status is required to appropriately assess how the compounding role of systemic racial bias may impact pain perception and treatment in said populations. 

## 6. Conclusions

Research in pediatric pain in the context of hypermobility disorders to date has focused on the incidence of musculoskeletal pain and related interventions. However, preliminary evidence suggests that the incidence of non-musculoskeletal pain (e.g., headaches, abdominal pain, gynecological complaints) are frequent complaints that require additional research to better understand and effectively treat. Appropriate interventions for musculoskeletal and non-musculoskeletal pain alike are crucial, given the described relationship between pain and quality of life in pediatric JHS and EDS populations. Additionally, as future research addresses the shortcomings in the present understanding of pain and intervention for such pain in the context of pediatric EDS, the consideration of sample construction (e.g., operationalization of EDS, and racial and sex diversity) is crucial to produce research that is appropriately representative and generalizable to all pediatric EDS patients. 

## Figures and Tables

**Table 1:** Summary of Pain Sites in Pediatric Hypermobility Syndromes.

Site of Pain	Authors	Populations Described
Musculoskeletal		
Back	Mato et al., 2008 [37]; Stern et al., 2017 [36]; Tran et al., in press [33]; Walter, 2014 [38]	hEDS, JHS, cEDS, vEDS
Neck	Mato et al., 2008 [37]; Tran et al., in press [33]; Walter, 2014 [38]	hEDS, JHS, cEDS
Shoulders	Brown and Stinson, 2004 [39]; Schubert-Hjalmarsson et al., 2012 [21]; Stern et al., 2017 [36]; Tran et al., in press [33]	hEDS, JHS, cEDS, vEDS,
Wrists	Brown and Stinson, 2004 [39]; Mato et al., 2008 [37]; Schubert-Hjalmarsson et al., 2012 [21]	hEDS, JHS
Hips	Mato et al., 2008 [37]	JHS
Knees	Brown and Stinson, 2004 [39]; Mato et al., 2008 [37]; Schubert-Hjalmarsson et al., 2012 [21]; Stern et al., 2017 [36]	hEDS, JHS, cEDS, vEDS,
Feet	Mato et al., 2008 [37]; Schubert-Hjalmarsson et al., 2012 [21]	hEDS, JHS
Small joints (toes, fingers)	Brown and Stinson, 2004 [39]; Moore, Tolo and Weiland, 1985 [40]	hEDS
Non-Musculoskeletal		
Headaches	Mato et al., 2008 [37]; Savasta et al., 2007 [42]; Tran et al., in press [33]; Walter, 2014 [38]	JHS, cEDS
Abdominal Pain	Mato et al., 2008 [37]; Pacey et al., 2015 [17]	hEDS, JHS
Dysmenorrhea	Hugon-Rodin et al., 2016 [43]; Hurst et al., 2014 [44]	hEDS, vEDS
Painful piezogenic pedal papules	Kahana et al., 1985 [45]; Poppe and Hamm, 2013 [46]	cEDS

Note: hEDS = hypermobility type EDS, JHS = joint hypermobility syndrome, cEDS = classical type EDS, vEDS = vascular type EDS.

**Table 2:** Summary of Quality of Life Correlates of Pain in Pediatric Hypermobility Syndromes.

Quality of Life Correlate	Sample Demographics (N, Illness Group)	Authors and Key Finding
Emotional Functioning	N = 34, hEDS	Tran et al., in press [33]: Anxiety, R = 0.50, Depression, R = 0.48
School Functioning	N = 54, JHS	Mato et al., 2008 [37]: Only 35% attended school full time
Physical Functioning	N = 54, JHS	Mato et al., 2008 [37]: Only 6% of children completed gym class activities
	N = 32, JHS	Engelbert et al., 2006 [84]: diminished peak maximal oxygen consumption as compared to those without JHS associated pain (Cohen’s d = 0.93)
	N = 125, JHS	Adib et al., 2005 [15]: 85% of children note exacerbation of pain with exercise
